# Diversity of RNA viruses in agricultural insects

**DOI:** 10.1016/j.csbj.2023.08.036

**Published:** 2023-09-03

**Authors:** Yu-Hua Qi, Zhuang-Xin Ye, Chuan-Xi Zhang, Jian-Ping Chen, Jun-Min Li

**Affiliations:** State Key Laboratory for Managing Biotic and Chemical Threats to the Quality and Safety of Agro-products, Key Laboratory of Biotechnology in Plant Protection of Ministry of Agriculture and Zhejiang Province, Institute of Plant Virology, Ningbo University, Ningbo 315211, China

**Keywords:** Virus discovery, RNA viruses, Agricultural insect pests, Beneficial insects

## Abstract

Recent advancements in next-generation sequencing (NGS) technology and bioinformatics tools have revealed a vast array of viral diversity in insects, particularly RNA viruses. However, our current understanding of insect RNA viruses has primarily focused on hematophagous insects due to their medical importance, while research on the viromes of agriculturally relevant insects remains limited. This comprehensive review aims to address the gap by providing an overview of the diversity of RNA viruses in agricultural pests and beneficial insects within the agricultural ecosystem. Based on the NCBI Virus Database, over eight hundred RNA viruses belonging to 39 viral families have been reported in more than three hundred agricultural insect species. These viruses are predominantly found in the insect orders of Hymenoptera, Hemiptera, Thysanoptera, Lepidoptera, Diptera, Coleoptera, and Orthoptera. These findings have significantly enriched our understanding of RNA viral diversity in agricultural insects. While further virome investigations are necessary to expand our knowledge to more insect species, it is crucial to explore the biological roles of these identified RNA viruses within insects in future studies. This review also highlights the limitations and challenges for the effective virus discovery through NGS and their potential solutions, which might facilitate for the development of innovative bioinformatic tools in the future.

## 1 Introduction

Viruses are the most abundant entities in the global biosphere. However, traditional research has primarily focused on viruses causing diseases in humans or economically important species [1]. Over the past decade, advancements in next-generation sequencing (NGS) technology and bioinformatics tools have unveiled the vast diversity of viruses, particularly highlighting the often-underestimated number of RNA viruses [2]. Recent metagenomic studies have revealed an astonishing abundance of RNA viruses in invertebrates, notably among insect species that have lived on earth for over 400 million years [3], [4], [5], [6]. Insects can be infected by two broad categories of viruses: arboviruses and insect/arthropod-specific viruses (ISVs) [7]. Arboviruses have the capability to infect both insects and vertebrates (or plants) and have been extensively studied as causative agents of overt diseases. In contrast, ISVs are unable to replicate in vertebrates and are restricted to infecting insect hosts. However, they may influence the transmission of arboviruses by vector insects [8], [9]. Recent unbiased metagenomic approaches have uncovered a remarkable diversity of ISVs, filling major gaps in our understanding of viral evolutionary history. Furthermore, numerous in vitro and in vivo studies have demonstrated the ability of ISVs to modulate pathogenic arboviruses [10].

While comprehensive reviews on the diversity of ISVs and their potential impact on arbovirus transmission have emerged in recent years [7], [8], [9], [11], [12], [13], these reviews have primarily focused on hematophagous insects. However, there is currently a lack of a review specifically addressing the viromes of agriculturally relevant insects. Therefore, the objective of this review is to provide a comprehensive overview of the diversity of RNA viruses in insects within the agricultural ecosystem. In the subsequent sections, we will present the RNA viromes reported in major agricultural insect orders and outline current limitations for virus discovery using NGS method.

## 2 Overview of RNA viruses in agricultural insects

**Fig. 1:**
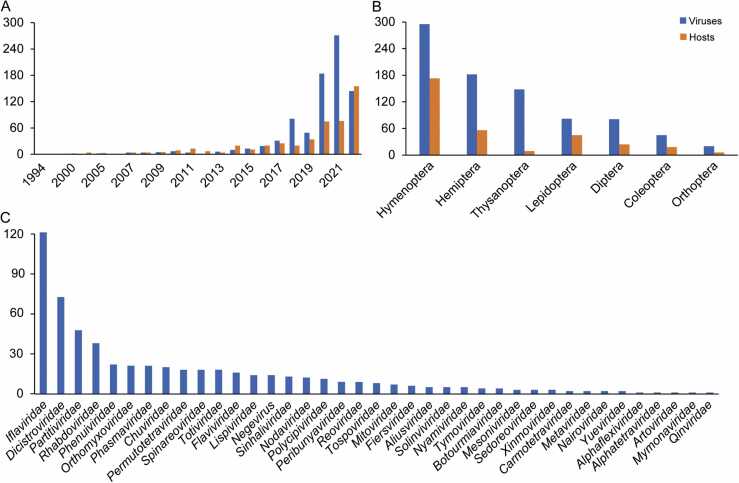
Diversity of RNA viruses in agricultural insects. (A) The increasing number of RNA viruses and agricultural insect hosts from 1994 to 2022. (B) The distribution of RNA viruses in agricultural insect host species across different orders. (C) The diversity of RNA viruses belonging to different families in agricultural insects.

**Fig. 2:**
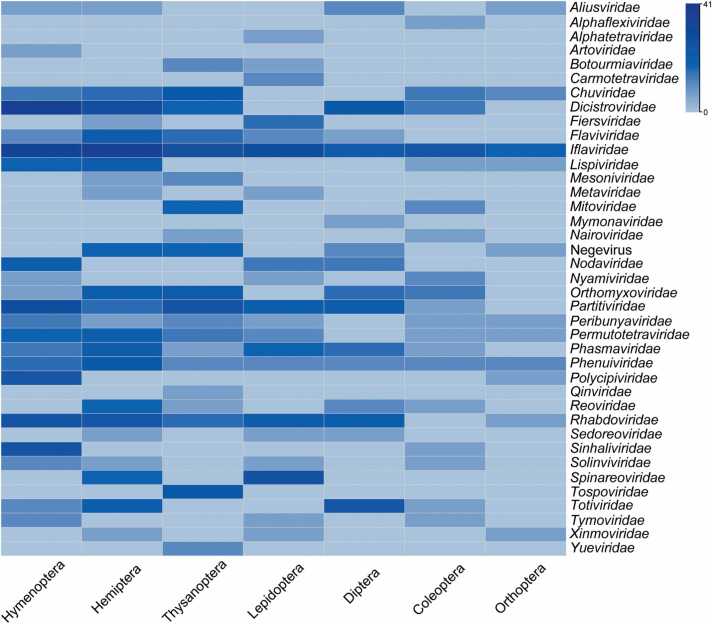
Diversity of RNA viruses in the seven orders of agricultural insects.

The number of RNA viruses associated with agricultural insects submitted to the NCBI Virus Database (https://www.ncbi.nlm.nih.gov/labs/virus) has significantly increased in recent years. This database is a community portal for viral sequence data collected from RefSeq, GenBank and other NCBI repositories, which is updated frequently following new viral sequence submissions. Although several defects were reported, such as incomplete viral genomes and uncertain hosts for some viruses, this database is still serving as the major viral reference resource for the growing community needs [14], [15], [16]. In the year 2022, approximately 144 viruses were submitted in association with 155 agricultural insect hosts (Fig. 1A). As of March 27, 2023, a total of 853 RNA viruses have been identified in 331 agricultural insect species (Supplementary File S1). These viruses are primarily distributed among the following insect orders: Hymenoptera (295 viral species), Hemiptera (182 viral species), Thysanoptera (148 viral species), Lepidoptera (82 viral species), Diptera (81 viral species), Coleoptera (45 viral species), and Orthoptera (20 viral species) (Fig. 1B and Fig. 2) (Supplementary File S1). Among these insect orders, Hymenoptera had the highest number of viruses, as well as their hosts, followed by Hemiptera (Fig. 1B and Supplementary Table S1). At the family level, the most abundant viral families identified in agricultural insects are *Iflaviridae*, *Dicistroviridae*, *Partitiviridae*, and *Rhabdoviridae*, with 121, 68, 46, and 39 virus species, respectively (Fig. 1C).

## 3 RNA viruses in agricultural insects of different orders

### 3.1 RNA viruses in Hymenoptera

Hymenoptera is a diverse order of insects of holometabolous species such as bees, wasps, hornets, sawflies, and ants. While the majority of Hymenoptera species serve as beneficial pollinators or act as parasitic or predatory insects, there is a minority of herbivorous pests that can impact agricultural crops [17], [18]. A total of 8153 viral sequences have been submitted, along with over 229 hosts from the order Hymenoptera, with a significant proportion of these viruses being discovered in bees. Among the 295 viruses discovered in 173 important hymenopteran insects, which are mainly pollinators such as bees and wasps, the *Iflaviridae* and *Dicistroviridae* families were the most abundant (Fig. 2, Supplementary Fig. S1 and Supplementary Table S1). Notably, more than one hundred viruses (119) were identified in a single bee species, *Apis mellifera*, underscoring the importance of viruses in the beekeeping industry.

Bees, primarily feeding on pollen and nectar, play a crucial role in crop pollination and the production of bee products valuable to humans. However, bees are facing significant risks from pathogenic viruses, which can cause colony losses and pose a serious threat to bee health. Recognizing the vital importance of bees in maintaining food security and biodiversity, extensive research has been conducted to explore viral diversity in bees [19], [20], [21], [22], [23], [24], [25], [26]. Early investigations into bee viruses date back to the 20th century. Two of the first identified bee viruses, Acute bee paralysis virus (ABPV) and Deformed wing virus (DWV), were found to be associated with colony death or collapse [19], [20], [27], [28], [29]. The application of metagenomics has played a pivotal role in the discovery of additional virus isolates and novel viruses in various bee species [22], [24], [26]. For instance, studies focused on *A. mellifera* (the Western honey bee) have revealed the presence of seven novel viruses in geographically distinct honey bee populations, indicating greater viral diversity within honey bee colonies than previously known [22]. Furthermore, a recent study in China characterized the prevalence and evolution of 23 novel honeybee viruses [26]. However, the spread of viruses in honey bees has become a growing concern, particularly with the emergence of the parasitic mite *Varroa destructor* and other contributing factors [29], [30], [31]. *Varroa* mites are a major driver of managed European honeybee colony losses worldwide and directly affect the feeding of immature bees. Additionally, *Varroa* mites have been confirmed as vectors of five debilitating viruses [32]. Moreover, metagenomic analysis of *Varroa*-free Australian honey bees has revealed a diverse virome within the order *Picornavirales*, suggesting a preference for infection by these viruses in honey bees and highlighting the potential impact of *Varroa* mites on the honey bee virome [23].

Wasps, particularly those of the *Vespula* genus, have been frequently reported to harbor a diverse range of RNA viruses [25], [33], [34], [35]. These social insects have become significant pests and predators in areas where they have been introduced [36]. In a comprehensive study, 68 novel and nine previously reported viral sequences were characterized in the transcriptomes of *V. vulgaris* colonies from both their native range (Belgium) and an invasive range (New Zealand) [34]. RNA viruses have also been identified in parasitic wasps, which are natural enemies of insect pests [33], [37], [38], [39]. Transcriptomic analysis of three wasp species, *Anisopteromalus calandrae*, *Lariophagus distinguendus*, and *Theocolax elegans*, known for parasitizing and controlling the rice weevil *Sitophilus oryzae*, a major insect pest of stored grains, led to the discovery of six novel RNA viruses [33]. Intriguingly, another study demonstrated that a novel negative-stranded RNA virus (family *Nyamiviridae*) infecting a parasitoid wasp (*Pteromalus puparum*) could influence the sex ratio of the host's offspring by decreasing the number of female offspring [39]. Additionally, novel RNA viruses were identified in the red imported fire ant, *Solenopsis invicta*, an extremely destructive invasive species [40], [41], [42]. In a recent investigation, nine new RNA viruses and six previously described Solenopsis viruses were discovered in *S. invicta* populations collected from its native range in Formosa, Argentina [42].

### 3.2 RNA viruses in Hemiptera

The Hemiptera order comprises numerous insect species that have significant negative impacts on agriculture. Some hemipteran insects, such as aphids and leafhoppers, pose a serious threat by feeding on plants and extracting sap, which leads to stunted growth, reduced yield, and even plant mortality [43]. Furthermore, certain hemipterans serve as vectors for viral diseases, acquiring viruses from infected plants and transmitting them to healthy ones, thereby causing severe crop diseases [44], [45]. Notable examples of important plant virus vectors include planthoppers, leafhoppers, and whiteflies. Statistical data indicates that a total of 1668 RNA viral sequences, representing 280 RNA viruses have been discovered in more than 92 hemipteran insects. Among these, 182 viruses have been found to infect 56 various agricultural pests. These viruses belong to diverse families or groups, with the *Iflaviridae* family being the most prevalent (41 viruses), followed by *Dicistroviridae* (15 viruses) and *Rhabdoviridae* (10 viruses) (Fig. 2, Supplementary Fig. S2 and Supplementary Table S1). Among the 56 agricultural pest insect species, *Bemisia tabaci* harbors the highest number of viral infections (21 viruses), followed by *Nilaparvata lugens* (17 viruses), and *Laodelphax striatellus* (12 viruses). Notably, *N. lugens* (nine families), *L. striatellus* (eight families), and *B. tabaci* (seven families) exhibit the greatest taxon diversity of RNA viruses. This could be attributed to extensive studies on these pests as significant agricultural pests and virus vectors. Furthermore, several viruses have also been identified in other common pests including aphids, leafhoppers, psyllids, and stinkbugs (Supplementary File S1) [46], [47], [48], [49], [50], [51], [52], [53], [54], [55], [56], [57], [58].

The whitefly *B. tabaci* is a globally significant agricultural pest [59]. In recent years, several RNA viruses belonging to various viral families associated with *B. tabaci* have been discovered [60], [61], [62], [63]. The first ISV of whiteflies belonging to the family *Dicistroviridae* was identified in 2019 [62], followed by the report of another novel dicistrovirus [61]. More recently, a systematic screening of the RNA virome in different cryptic species of whiteflies led to the discovery of 20 new RNA viruses. These viruses belong to multiple orders/families/groups, including *Lispiviridae*, *Nidovirales*, *Flaviviridae*, Negevirus, *Virgaviridae*, *Picornavirales*, *Orthomyxoviridae*, and *Totiviridae* [60].

ISVs of the three rice planthoppers, *N. lugens*, *L. striatellus*, and *Sogatella furcifera* have also been explored due to their significant importance in rice production [64]. Nilaparvata lugens reovirus (NLRV) was the first ISV isolated from rice planthoppers (*N. lugens*) in 1991 [65], followed by Himetobi P virus (HiPV), originally identified from *L. striatellus* [66], and a novel satellite virus particles purified from *N. lugens* [67]. Subsequently, more studies have reported several novel RNA viruses through transcriptome sequencing, including iflaviruses, a cripavirus, and an orthomyxovirus in *N. lugens* [5], [68], [69], [70], a hepe-like virus, iflaviruses, and totiviruses in *S. furcifera* [71], [72], [73], and an iflavirus and a fijivirus in *L. striatellus* [74], [75].

In addition to *B. tabaci* and the three rice planthoppers, novel RNA viruses have also been identified in other notable hemipteran pests of various crops such as aphids, leafhoppers, whiteflies (other than *B. tabaci*), psyllids, mealybugs, stinkbugs, and bean bugs. The NCBI Virus Database contains a total of 38 viruses from 18 leafhopper species, 37 viruses from 15 aphid species, nine viruses from four other whitefly species and 16 viruses from five stinkbug species.

Most of the ISVs in different leafhopper species are currently reported sporadically. For example, a cripavirus in *Homalodisca coagulata* [76], iflaviruses in *Euscelidius variegatus* [77]*, Psammotettix alienus* [78], *Nephotettix cincticeps* [79], and *Recilia dorsalis* [53]. Furthermore, a chuvirus and a reovirus were identified in *P. alienus* [80], [81], and an iflavirus and a nido-like virus were discovered in *Cicadella viridis* [51]*.* In a recent study, nine novel RNA viruses were identified in *Scaphoideus titanus*, belonging to seven viral clades (Picorna-Calici, Permutotetra, Bunya-Arena, Reo, Partiti-Picobirna, Luteo-Sobemo and Toti-Chryso) [82].

Aphids, well-known as vectors of many plant viruses, also have intricate relationships with symbiotic microorganisms [50]. Virus-like sequences related to nege/kita-, flavi-, tombus-, phenui-, mononega-, narna-, chryso-, partiti-, and luteoviruses were discovered in aphids infesting barley plants in a field in Japan [50]. Recently, an RNA-seq virome study uncovered 18 bunyaviruses from 10 aphid species. Notably, Aphid bunyavirus 1 (ABV-1) infected and replicated in all 10 aphid species, indicating ABV-1 has a wide host range [83]. Additionally, Aphid lethal paralysis virus (ALPV) was identified in various aphid host species, including *Acyrthosiphon pisum*, *Aphis fabae*, *A. glycines*, and *A. nerii* [84], [85], [86]. A flavivirus was also reported from *Macrosiphum euphorbiae* [87], and a novel ISV (Aphis Glycines Virus 2, AGV-2) with a unique genome structure was discovered in *A. glycines*. The RNA-dependent RNA polymerase (RdRp) of AGV-2 is similar to insect tetraviruses, while the capsid protein (CP) shares structural similarities with plant sobemoviruses [46].

Whiteflies other than *B. tabaci* have also been found to harbor ISVs, such as a chuvirus and an arlivirus in *Trialeurodes vaporariorum* [5], [6]. Furthermore, a comprehensive survey revealed diverse virus-like sequences in the Asian citrus psyllid (*Diaphorina citri*), which is a key insect associated with citrus production worldwide. Novel viral sequences belonging to the *Picornavirales*, *Reoviridae*, and *Bunyaviridae*, as well as an unclassified RNA virus were identified in *D. citri* [54], [55]. A picorna-like virus was also found in the potato/tomato psyllid (*Bactericera cockerelli*) [56]. Among mealybugs, dicistroviruses were reported in *Phenacoccus solenopsis* and *Planococcus ficus* [88], [89], and a rhabdovirus was found in *P. citri* [5]. Additionally, ISVs have been discovered in several stinkbug species, including a chuvirus in *Nezara viridula* [57], a novel arlivirus in *Erthesina fullo* [58], a picorna-like virus in *Arma chinensis* [90], and seven new viral sequences from *Halyomorpha halys* [91]. In *Riptortus pedestris*, a bean bug that causes significant damage to leguminous plants, a total of five ISVs were identified [92], [93], [94]. Apart from the ISVs mentioned above, more ISVs have been found in several other hemipteran species related to agriculture. These include an iflavirus in the spotted lanternfly (*Lycorma delicatula*) [95], an unclassified ISV in the tomato bug (*Nesidiocoris tenuis*) [96], and an iflavirus in the tarnished plant bug (*Lygus lineolaris*) [97].

### 3.3 RNA viruses in Thysanoptera

Thysanoptera, commonly known as thrips, encompass a wide range of insect species that pose a significant threat to crops. These pests directly feed on plants and can transmit plant viruses, resulting in substantial damage to agricultural production [98]. Currently, most of the RNA viruses discovered in Thysanoptera are found in pest thrips species. The NCBI Virus Database has documented a total of 225 RNA viral sequences, including 148 RNA viruses from nine agricultural thrips species. Notably, the most abundant RNA virus families in Thysanoptera are *Iflaviridae* (14 viruses) and *Partitiviridae* (12 viruses) (Fig. 2, Supplementary Fig. S3 and Supplementary Table S1).

Among the thrips species studied for virome analysis, three species stand out in terms of viral diversity. *Neohydatothrips variabilis*, also known as soybean thrips, has gained attention for its role as an efficient vector of soybean vein necrosis virus, which causes severe necrotic symptoms in susceptible soybean plants [99]. Metatranscriptome analysis of *N. variabilis* revealed a broad range of virus-like sequences, including 155 novel ones associated with ISVs and other viruses that are similar to plant and fungus-infecting viruses. These findings suggest that *N. variabilis* can acquire viruses from various host plants and potentially transmit them to soybean crops, highlighting the underestimated importance of their role in virus dissemination [100]. Additionally, *Frankliniella occidentalis*, commonly known as western flower thrips, and *Thrips tabaci,* known as onion thrips, significantly impact horticultural crops through direct damage and efficient transmission of tomato spotted wilt virus and iris yellow spot virus, respectively [101], [102]. A comprehensive study investigating thrips populations in Italy and the United States identified a total of 41 viruses, including 14 viruses in *F. occidentalis*, 17 viruses in *T. tabaci*, and one in *N. variabilis* [103]. These systematic studies have provided valuable insights into the viral diversity in thrips. However, the virome in thrips remains inadequately examined, and more research is needed to better understand the complex interactions between thrips and the multiple viruses present in agricultural systems.

### 3.4 RNA viruses in Lepidoptera

Lepidoptera, the second largest order of insects, has a wide distributed and the highest species diversity in tropical regions [104]. While the domestic silkworm (*Bombyx mori*) is a well-known economically important insect, the larvae of most other species pose threats to various cultivated plants, with larger-bodied species often consuming leaves or boring into branches. To date, approximately 735 RNA viral sequences (representing 107 RNA viruses) have been reported in Lepidoptera with more than 57 hosts species, including 82 RNA viruses associated with agricultural pests from 45 different species (Supplementary File S1). Although viruses in moths have been studied more extensively compared to butterflies, only one virus has been found in most moth species. Among the identified RNA viruses, nine have been discovered in *Chilo suppressalis* and eight in *Thaumetopoea pityocampa*. The families *Iflaviridae* (16 viruses) and *Spinareoviridae* (13 viruses) are more prevalent among the identified RNA viruses in Lepidoptera (Fig. 2, Supplementary Fig. S4 and Supplementary Table S1).

Several studies have identified five RNA viruses in cultured cells of *B. mori* or *B. mori* pupa, including an iflavirus and a macula-like virus [105], [106]. *T. pityocampa*, commonly known as the pine processionary moth, is a significant pine pest found in forests across the Mediterranean, Central Europe, the Middle East, and North Africa. Three studies on *T. pityocampa* have revealed RNA viruses belonging to different viral families, including *Spinareoviridae*, *Iflaviridae*, *Permutotetraviridae*, *Partitiviridae*, *Flaviviridae*, and *Rhabdoviridae*. These viruses could be potential candidates for the development of novel biocontrol strategies [107], [108], [109]. The pink bollworm, *Pectinophora gossypiella*, a major cotton pest, has recently been found to harbor four novel viruses in its pheromone glands [110]. Furthermore, several moth species have been reported to host multiple viruses. This includes the discovery of three novel bunyaviruses, two novel rhabdoviruses, and one novel nyamivirus found in moths in Washington state [111]. Additionally, other studies have discovered single novel viruses in specific insect hosts, such as iflaviruses in *Antheraea mylitta* [112], *A. pernyi* [113], and *Helicoverpa armigera* [114], a bunyavirus in *Euproctis pseudoconspersa* [115], cypoviruses in *Biston robustus* [116] and *Choristoneura occidentalis* [117], partitiviruses in *Homona magnanima* [118] and *Spodoptera exempta* [119], [120], a rhabdovirus in *S. frugiperda* [121], [122], and a picorna-like virus in *H. armigera* [123]. Apart from the viruses discovered in moths, four RNA viruses have been found in four butterfly species, including viruses belonging to the families *Xinmoviridae*, *Iflaviridae*, and *Nodaviridae* found in butterflies of *Colias croceus* [5], *Opsiphanes invirae* [124] and *Urbanus proteus* [5], and *Pieris rapae* [125], respectively.

### 3.5 RNA viruses in Diptera

The order Diptera includes insects such as flies, mosquitoes, and midges. Mosquitoes and midges are particularly important due to their ability to transmit viral diseases, while certain groups of fly larvae, such as tephritid fruit flies, leaf-mining flies, and fruit flies, are considered agricultural pests [126]. This review primarily focuses on RNA viruses associated with agricultural pests within the order Diptera. According to the NCBI Virus Database, a total of 1561 RNA viral sequences associated with 81 Diptera hosts were reported. And 81 RNA viruses have been identified in agricultural pests from 24 different host species (Fig. 2, Supplementary Fig. S5 and Supplementary Table S1). Notably, there were 17 RNA viruses identified in *Drosophila suzukii*, nine in *Bactrocera dorsalis*, and eight in *B. tryoni* (Supplementary File S1).

*D. suzukii* is an invasive pest that infests ripening fruit, causing significant economic losses. A metatranscriptomic study was conducted to identify viruses infecting this fly in both its native (Japanese) and invasive (British and French) ranges. This study revealed over 10 novel RNA viruses, including members of the *Picornavirales*, *Mononegavirales*, *Bunyavirales*, *Nidovirales*, *Chuviridae*, *Nodaviridae*, *Tombusviridae*, and *Reoviridae*. Additionally, 18 previously described viruses from other *Drosophila* species were detected in wild *D. suzukii* populations [127]. Furthermore, a pathogenic iflavirus has also been reported in *D. suzukii* [128]. More recently, a study identified 39 viral sequences from samples of 12 fruit fly species (family Tephritidae, genera *Bactrocera* and *Zeugodacus*) that are destructive to fruits and vegetables. Specifically, they found four RNA viral sequences in *B. dorsalis*, four RNA viral sequences in *B. correcta*, one RNA viral sequence in *B. minax*, five RNA viral sequences in *Z. tau*, six RNA viral sequences in *Z. cucurbitae*, and 19 RNA viral sequences in seven other *Bactrocera* species. These RNA viruses represented various viral taxa including *Dicistroviridae*, negev-like virus clade, *Solemoviridae*, *Narnaviridae*, *Nodaviridae*, *Iflaviridae*, *Orthomyxoviridae*, *Bunyavirales*, *Partitiviridae*, and *Reoviridae* [129]. Interestingly, eight novel RNA viruses were also reported in *B. dorsalis* from a laboratory colony, including four positive-strand RNA viruses, two negative-strand RNA viruses, and two double-stranded RNA viruses [130]. Additionally, recent research expanded the medfly RNA virome to include 13 viruses, including two novel positive ssRNA viruses and two novel dsRNA viruses [131]. In a comprehensive analysis of RNA virome composition in tephritid fruit flies, which are among the most devastating pests of horticulture, 34 putative viruses belonging to eight RNA virus families were found [132]. Moreover, RNA viruses have also been reported in other agricultural dipterans, including four novel viruses in *Atherigona orientalis* [3], five novel viruses in *Melophagus ovinus* [133], an iflavirus and negeviruses in *Glossina morsitans morsitans* [134], and reo-like, bunya-like, partiti-like and orthomyxo-like viruses in blow flies (*Calliphora augur* and *C. vicina*) [135].

### 3.6 RNA viruses in Coleoptera

The order Coleoptera, commonly known as beetles, is the largest order within the class Insecta. Beetles include species that can be either significant pests or beneficial insects in agricultural, forestry, fruit trees, and horticulture ecosystems [136]. For instance, grain weevils like *S. oryzae* are important agricultural pests, while the Australian ladybird beetle *Coelophora inaequalis* from the family Coccinellidae, serves as a valuable natural enemy insect. Despite the vast diversity of beetle species, the number of RNA viruses discovered and associated with them remains limited. Currently, there have been 210 submitted RNA viral sequences (representing 107 RNA viruses) identified in 33 beetle hosts, including 45 viruses found in 18 agricultural insect hosts (Supplementary File S1). The most frequently identified RNA viruses belong to the *Iflaviridae* family, comprising a total of nine viruses (Fig. 2, Supplementary Fig. S6 and Supplementary Table S1).

One previous study investigated the virome in larvae of alfalfa weevils (*Hypera postica*), which are major herbivorous pests that feed on legumes. This study identified and characterized five novel RNA viruses associated with weevils [137]. Additionally, various studies have reported RNA viruses associated with agricultural insects of the order Coleoptera. These include the discovery of bee-associated viruses in *Aethina tumida* [138], several novel RNA viruses in *Dermolepida albohirtum* [139], an orthomyxovirus in *Ips typographus*, a chuvirus and a phenuivirus in *Larinus minutus* [5], and an iflavirus in *Aulacophora lewisii* [140].

### 3.7 RNA viruses in Orthoptera

Orthoptera insects, including grasshoppers, are primarily phytophagous species, and many of them are significant agricultural pests known for their ability to swarm in large numbers, causing extensive damage to crops and posing a major economic threat to agriculture [141]. However, the number of RNA viruses discovered and reported in Orthoptera is relatively limited. Currently, 135 RNA viral sequences (representing 57 RNA viruses) have been submitted to the NCBI Virus Database for 15 orthopterans, with 20 of them associated with agricultural pests from six different host species (Fig. 2, Supplementary Fig. S7 and Supplementary Table S1).

A recent study conducted a comprehensive analysis of viral communities in 45 grasshopper species, including many major agricultural pests. This study represents the first extensive exploration of RNA viruses in grasshoppers, providing a valuable dataset for the understanding of the natural pathogens infecting grasshoppers [142]. Another study identified and characterized a novel pathogenic reovirus called Acrididae reovirus (ARV) in the grasshopper *Locusta migratoria*. ARV was found to cause mortality, impair ovary development, and reduce fecundity in the host insect, suggesting its potential application as a biological control agent against grasshopper pests [143]. Furthermore, an RNA virome analysis of the pink-winged grasshopper *Atractomorpha sinensis* revealed four novel RNA viruses within the host, including a nege-like virus, an iflavirus, a chu-like virus, and an ollusvirus [144].

## 4 Limitations of NGS method for Insect Virus Discovery

The advent and development of NGS technology have propelled rapid and significant progress in the discovery of RNA viruses in agricultural insects, particularly in uncovering covert viruses and unveiling previously uncharacterized viral groups [145]. NGS technology enables the sequencing of complex mixtures of genetic material, allowing for cost-effective virus detection even when viral titers are low within infected hosts or tissues [146], [147]. However, despite the remarkable progress achieved in virus discovery through NGS, it still faces important challenges and limitations [2].

Given the inherent nature of indiscriminately sequencing viral contaminants alongside actual viruses presented in the sample, prudence is imperative when interpreting the results derived from NGS. Viral contaminants can be introduced across the various stages of sample preparation, ranging from sample collection and RNA extraction to the sequencing [135], [148], [149], [150]. Therefore, careful consideration of experimental design is essential to avoid potential sources of contamination [2]. When analyzing metagenomic data, it is possible that some assembled contigs with sequence similarity to viral sequences can potentially lead to false-positive detections, such as endogenous viral elements (EVEs). EVEs are genetic sequences derived from DNA or RNA viruses that have integrated into host genomes [151], [152], [153], [154]. Thus, virus species exhibiting noticeable abnormalities should be treated with caution. Whenever possible, duplicated experiments should be conducted for comprehensive comparisons. In the case of EVEs, their elimination can be achieved through comparison with the host genome. If necessary, the presence of identified viruses in the original samples need to be verified using Reverse-Transcription PCR [2], [155].

Besides the potential to generate false-positive viral sequences, the analysis of NGS data sometimes results in identification of viral sequences that don’t belong to the insects themselves. Instead, these viral sequences may actually result from host microbes (such as gut microbes or parasites carrying their own viruses), dietary components (e.g., plant or animal material), or other contaminants. Currently, it was estimated that nearly 40% of viral nucleotide records in GenBank do not have host information (excluding human viruses) [15]. Therefore, several strategies can be employed to accurately identify the most likely hosts for the newly detected viruses. These include the sampling of individual insect host or specific tissue, conducting phylogenetic analyses and utilizing well-defined host-virus associations. In addition, the analysis of virus-derived small RNA (sRNA) can also provide valuable insights to help define the true hosts [156].

Furthermore, the process of virus identification also faces technical challenges. Currently, the identification of viral sequences from NGS data mostly relied on the sequence-to-sequence comparisons with conserved viral nucleotide or protein sequences. This approach is typically effective only for novel viruses that share at least 30–40% amino acid identity with known viruses [157], which hampers the successful identification of novel viruses with highly divergent RdRPs using BLAST-based sequence similarity searches [158]. Therefore, methods based on profile alignment like Hidden Markov Models (HMMs) are also important for the discovery of new viruses with highly divergent RdRPs compared to the known viruses [157]. In addition, the development of innovative bioinformatics tools is crucial to accurately discern viruses that exhibit limited or no sequence similarity to known viruses. Recently, advancements in artificial intelligence (AI) have proven to be remarkably effective [159], [160], [161]. The application of deep learning algorithms in analyzing metagenomic data has shown promising results, representing a significant advancement in the field of virus discovery [162], [163], [164], [165].

## 5 Conclusions and future directions

Despite early research primarily focusing on viruses associated with vertebrate diseases, recent advances in metagenomics have led to the discovery of numerous ISVs in agricultural insects. It is now evident that insects harbor a diverse range of RNA viruses, with members belonging to at least 39 viral families or groups. These findings have significantly enhanced our understanding of viral diversity within agricultural insects. Experimental studies have also shown that ISVs exhibit host restrictions in arthropod vectors, distinguishing them from vector-borne viruses that can infect both vertebrate/plant and invertebrate hosts [7]. Phylogenetic analyses have revealed close relationships between many ISVs and vector-borne viruses, suggesting their potential influence on the transmission of arboviruses [8].

To gain a comprehensive understanding of ISVs in agricultural insects, it is crucial to explore viral diversity in a wide range of agriculturally important insects. While extensive research has been conducted on viral diversity in certain agricultural insects such as the western honey bee *A. mellifera*, soybean thrips *N. variabilis*, whitefly *B. tabaci*, and grasshopper *L. migratoria* [22], [26], [100], [166], further investigations are needed to explore the viromes of other agriculturally significant insects. For example, only one RNA virus has been reported in important pests like the fall armyworm (*S. frugiperda*) [120], red flour beetle (*T. castaneum*), and diamondback moth (*Plutella xylostella*). Furthermore, among beneficial insects, only three RNA viruses have been identified in ladybugs and syrphid flies, respectively [5], [167], [168]. Additionally, the viral diversity in agriculturally significant orthopterans, such as mantises, locusts, and katydids, is still understudied. Therefore, further investigations are necessary to explore the RNA viromes of these agriculturally significant insects.

Besides the discovery of numerous ISVs, our current understanding of their biology is limited. Most insect viruses have been identified through metagenomics, and their biology is mostly known from genomic sequences. While the majority of ISVs do not appear to significantly impact their hosts, it is important to validate their presumed abilities in infected host species. Further research should focus on the ecological distribution of these viruses in natural environments, their impact on host homeostasis, and their effect on vector competence. Several studies have already been conducted in these areas. For example, various ISVs have been implicated in the collapse and death of bee colonies [28]. A member of the viral family *Nyamiviridae* was found to influence offspring sex ratio by decreasing the number of female offspring in its parasitoid host [39]. Additionally, a partitivirus was shown to cause mortality in male *H. magnanima* during late development stages [118]. Recent findings have also demonstrated that ISVs can alter the vector competence of insects. For instance, iflaviruses in *S. exigua* were found to increase susceptibility to nucleopolyhedrovirus (NPV) disease [169]. Furthermore, a recent study discovered that arboviruses and symbiotic viruses cooperate to hijack insect sperm-specific proteins for paternal transmission [170]. These findings significantly enhance our understanding of the complex interactions between ISVs, arboviruses, and their insect hosts, providing valuable insights for potential prevention and control strategies.

Exploring the potential applications of ISVs in the prevention and control of vector-borne diseases is an important area of future research. The unique characteristics of ISVs, such as their limited host range, make them attractive candidates for biological control, prevention of vector-borne diseases, vaccine development, and diagnostics. Progress has already been achieved with insect-specific DNA viruses [171], [172]. Recent discoveries have shown the potential of RNA viruses for pest control, with a new cypovirus emerging as a potential agent for controlling lepidopteran pests [173]. Moreover, novel partiti-like viruses have shown resistance to NPV in their normal lepidopteran host *S. exempta*, but they appear to be deleterious in a novel pest host *S. frugiperda*, hinting at a possible pest management strategy through artificial host-shift of novel viruses [119]. Additionally, novel insect-specific eilat virus-based chimeric vaccine candidates have demonstrated durable, mono- and multivalent, single-dose protection against lethal alphavirus challenges [174], [175].

However, the advancement of research on insect viruses still faces technical challenges. Establishing cell lines suitable for studying insect viruses is a critical task that will provide more tools and resources for virus research [13]. Furthermore, the development of bioinformatics tools plays a vital role in advancing ISV research. Continued efforts to develop and refine these tools will greatly enhance our ability to characterize and study ISVs. Another important area of research is investigating the interactions between ISVs and other insect symbionts/pathogens. Understanding how these different pathogens and symbionts interact within the insect host is crucial for comprehending their roles in the transmission of vector-borne diseases. Interdisciplinary collaboration is essential to overcome these challenges and make significant progress. This collaborative effort will lead to comprehensive insights into the biology, ecology, and applications of ISVs.

## Author statement

All of the authors declare that this manuscript is original and has not been published before and is not currently, being considered for publication elsewhere. We confirm that the manuscript has only one author and that there are no other persons who, satisfied the criteria for authorship and are not listed. J.L. will be the responsible for communicating with the editor about progress, submissions of revisions and final approval of proofs.

## Declaration of Competing Interest

The authors whose names are listed immediately below certify that they have NO affi liations with or involvement in any organization or entity with any fi nancial interest (such as honoraria; educational grants; participation in speakers’ bureaus; membership, employment, consultancies, stock ownership, or other equity interest; and expert testimony or patent-licensing arrangements), or non-fi nancial interest (such as personal or professional relationships, affi liations, knowledge or beliefs) in the subject matter or materials discussed in this manuscript.

## Appendix A Supplementary material

.

Supplementary Fig. S1. Diversity of RNA viruses in Hymenoptera.

.

Supplementary Fig. S2. Diversity of RNA viruses in Hemiptera.

.

Supplementary Fig. S3. Diversity of RNA viruses in Thysanoptera.

.

Supplementary Fig. S4. Diversity of RNA viruses in Lepidoptera.

.

Supplementary Fig. S5. Diversity of RNA viruses in Diptera.

.

Supplementary Fig. S6. Diversity of RNA viruses in Coleoptera.

.

Supplementary Fig. S7. Diversity of RNA viruses in Orthoptera.
