# Supplementary material for: Diversity of RNA viruses in agricultural insects
Source: Comput Struct Biotechnol J. 2023 Sep 3;21:4312–21. doi: 10.1016/j.csbj.2023.08.036 (PMC10497914; doi:10.1016/j.csbj.2023.08.036)
Supplement: Supplementary file 1 — Supplementary material. [file mmc1.docx]

**Supplemental Table S1. The number of RNA viruses in the seven orders of agricultural insects.**

| **Insect order/**  **Virus family** | **Hymenoptera** | **Hemiptera** | **Thysanoptera** | **Lepidoptera** | **Diptera** | **Coleoptera** | **Orthoptera** |
| --- | --- | --- | --- | --- | --- | --- | --- |
| *Aliusviridae* | 1 | 1 | 0 | 0 | 2 | 0 | 1 |
| *Alphaflexiviridae* | 0 | 0 | 0 | 0 | 0 | 1 | 0 |
| *Alphatetraviridae* | 0 | 0 | 0 | 1 | 0 | 0 | 0 |
| *Artoviridae* | 1 | 0 | 0 | 0 | 0 | 0 | 0 |
| *Botourmiaviridae* | 0 | 0 | 2 | 1 | 0 | 0 | 0 |
| *Carmotetraviridae* | 0 | 0 | 0 | 2 | 0 | 0 | 0 |
| *Chuviridae* | 3 | 4 | 8 | 0 | 0 | 3 | 2 |
| *Dicistroviridae* | 37 | 15 | 5 | 0 | 8 | 3 | 0 |
| *Fiersviridae* | 0 | 1 | 0 | 4 | 0 | 0 | 0 |
| *Flaviviridae* | 2 | 7 | 4 | 2 | 1 | 0 | 0 |
| *Iflaviridae* | 29 | 41 | 14 | 16 | 7 | 9 | 5 |
| *Lispiviridae* | 5 | 7 | 0 | 0 | 0 | 1 | 1 |
| *Mesoniviridae* | 0 | 1 | 2 | 0 | 0 | 0 | 0 |
| *Metaviridae* | 0 | 1 | 0 | 1 | 0 | 0 | 0 |
| *Mitoviridae* | 0 | 0 | 5 | 0 | 0 | 2 | 0 |
| *Mymonaviridae* | 0 | 0 | 0 | 0 | 1 | 0 | 0 |
| *Nairoviridae* | 0 | 0 | 1 | 0 | 0 | 1 | 0 |
| Negevirus | 0 | 5 | 5 | 0 | 2 | 0 | 1 |
| *Nodaviridae* | 6 | 0 | 0 | 3 | 3 | 0 | 0 |
| *Nyamiviridae* | 1 | 0 | 0 | 1 | 0 | 2 | 0 |
| *Orthomyxoviridae* | 1 | 6 | 7 | 0 | 4 | 3 | 0 |
| *Partitiviridae* | 16 | 4 | 12 | 6 | 7 | 1 | 0 |
| *Peribunyaviridae* | 3 | 1 | 2 | 1 | 0 | 1 | 1 |
| *Permutotetraviridae* | 5 | 6 | 3 | 2 | 0 | 1 | 1 |
| *Phasmaviridae* | 3 | 7 | 1 | 5 | 4 | 1 | 0 |
| *Phenuiviridae* | 4 | 8 | 2 | 2 | 2 | 2 | 2 |
| *Polycipiviridae* | 10 | 0 | 0 | 0 | 0 | 0 | 1 |
| *Qinviridae* | 0 | 0 | 1 | 0 | 0 | 0 | 0 |
| *Reoviridae* | 0 | 5 | 1 | 0 | 2 | 1 | 0 |
| *Rhabdoviridae* | 11 | 10 | 4 | 7 | 6 | 0 | 1 |
| *Sedoreoviridae* | 0 | 1 | 0 | 1 | 1 | 0 | 0 |
| *Sinhaliviridae* | 12 | 0 | 0 | 0 | 0 | 1 | 0 |
| *Solinviviridae* | 2 | 1 | 0 | 1 | 0 | 1 | 0 |
| *Spinareoviridae* | 0 | 5 | 0 | 13 | 0 | 0 | 0 |
| *Tospoviridae* | 0 | 0 | 8 | 0 | 0 | 0 | 0 |
| *Totiviridae* | 2 | 6 | 0 | 0 | 9 | 1 | 0 |
| *Tymoviridae* | 2 | 0 | 0 | 1 | 0 | 1 | 0 |
| *Xinmoviridae* | 0 | 1 | 0 | 1 | 0 | 0 | 1 |
| *Yueviridae* | 0 | 0 | 2 | 0 | 0 | 0 | 0 |
| Unclassified | 139 | 38 | 59 | 11 | 22 | 9 | 3 |
| Total | 295 | 182 | 148 | 82 | 81 | 45 | 20 |
