# Supplementary figures and images for: Diversity of RNA viruses in agricultural insects
Source: Comput Struct Biotechnol J. 2023 Sep 3;21:4312–21. doi: 10.1016/j.csbj.2023.08.036 (PMC10497914; doi:10.1016/j.csbj.2023.08.036)

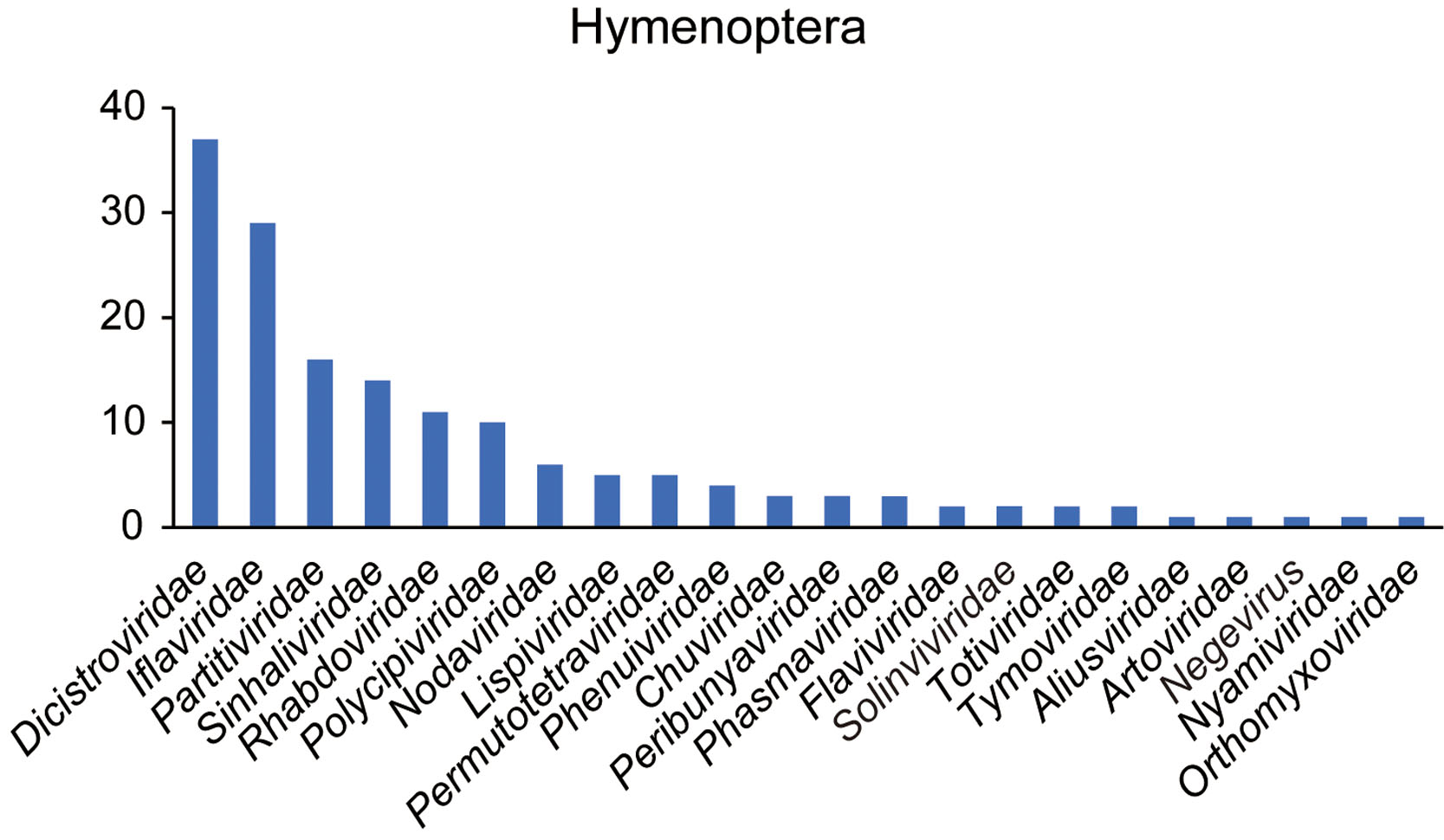

Supplement: Supplementary file 2 — Supplementary Fig. S1. Diversity of RNA viruses in Hymenoptera. [file mmc2.jpg]

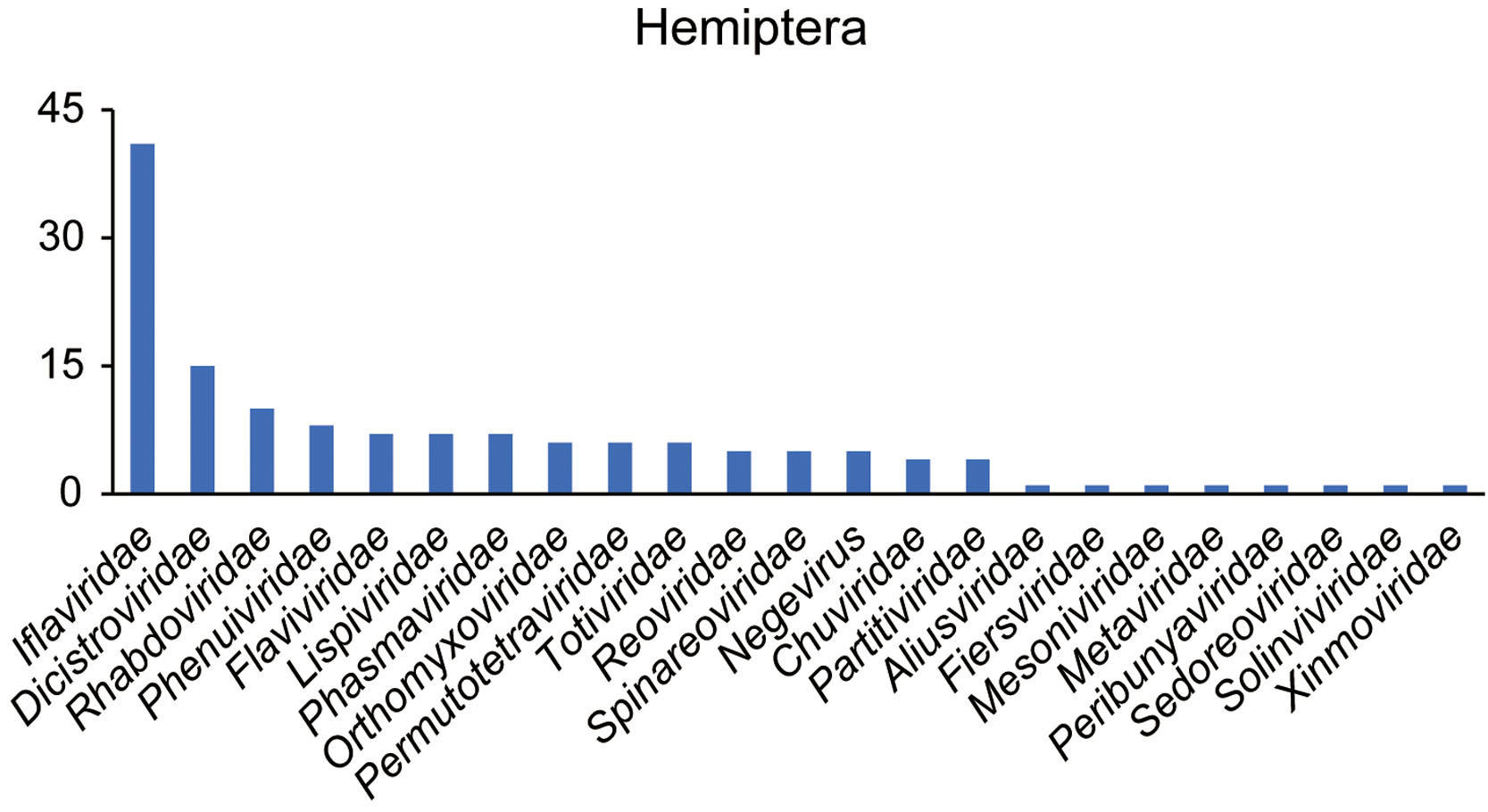

Supplement: Supplementary file 3 — Supplementary Fig. S2. Diversity of RNA viruses in Hemiptera. [file mmc3.jpg]

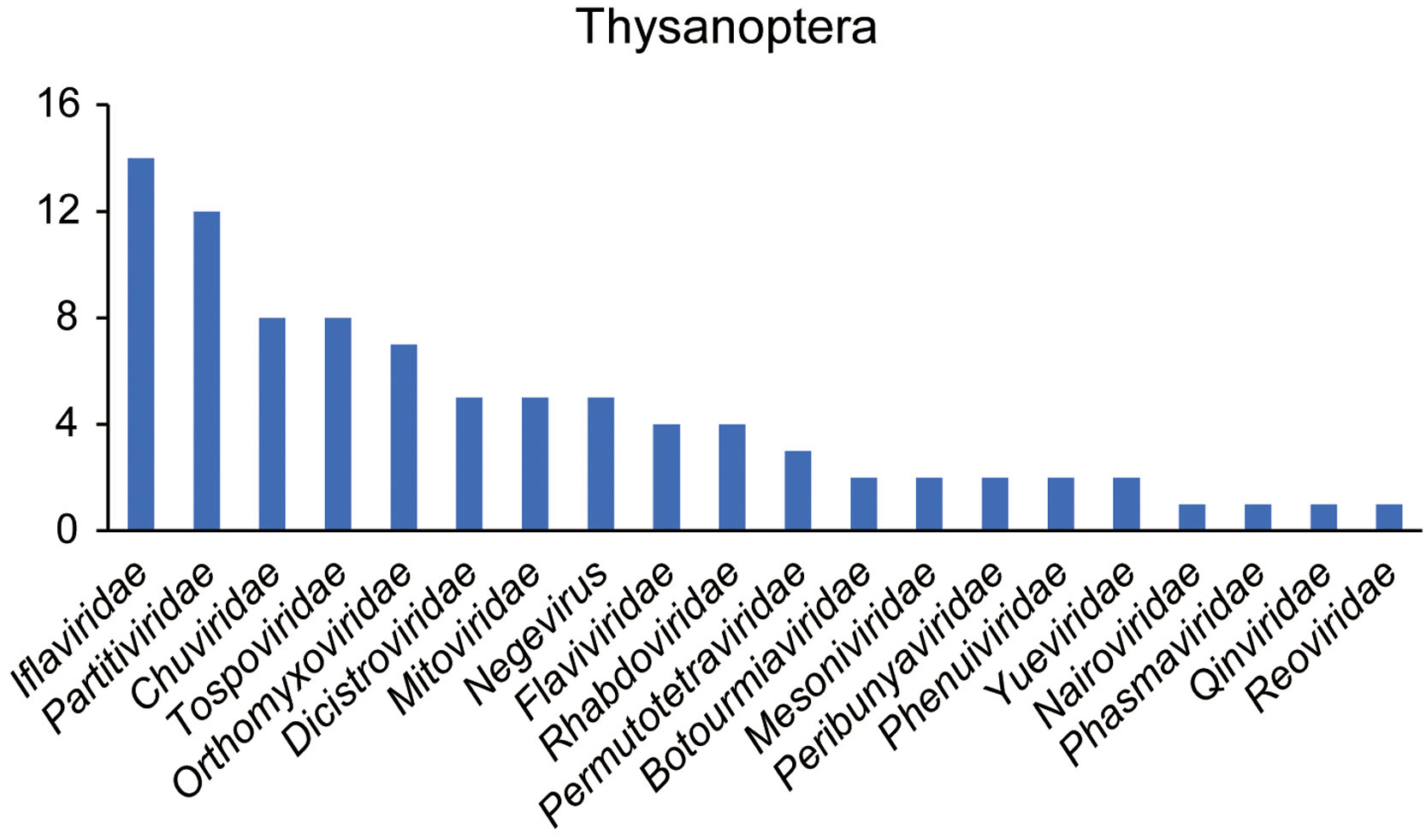

Supplement: Supplementary file 4 — Supplementary Fig. S3. Diversity of RNA viruses in Thysanoptera. [file mmc4.jpg]

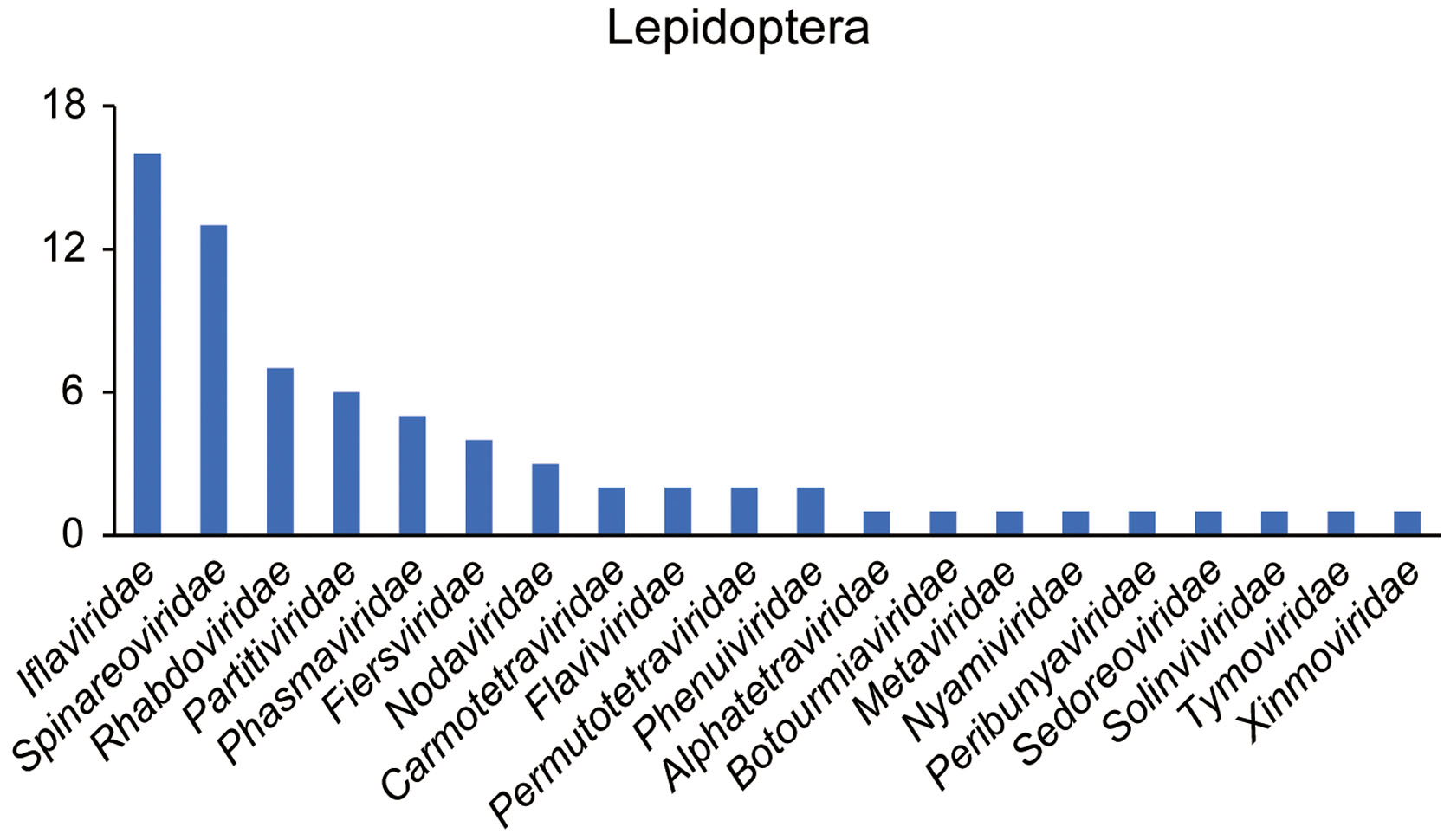

Supplement: Supplementary file 5 — Supplementary Fig. S4. Diversity of RNA viruses in Lepidoptera. [file mmc5.jpg]

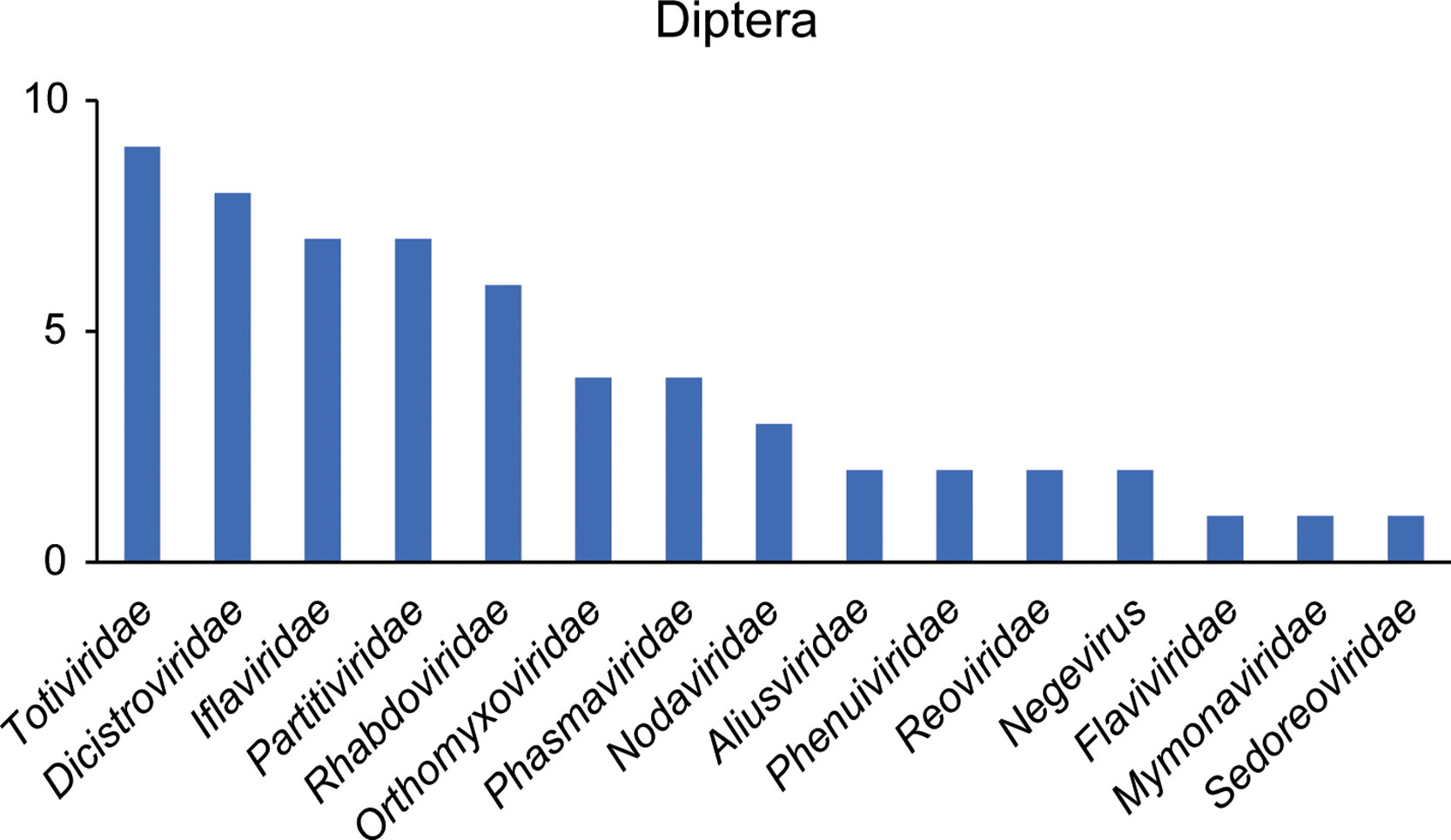

Supplement: Supplementary file 6 — Supplementary Fig. S5. Diversity of RNA viruses in Diptera. [file mmc6.jpg]

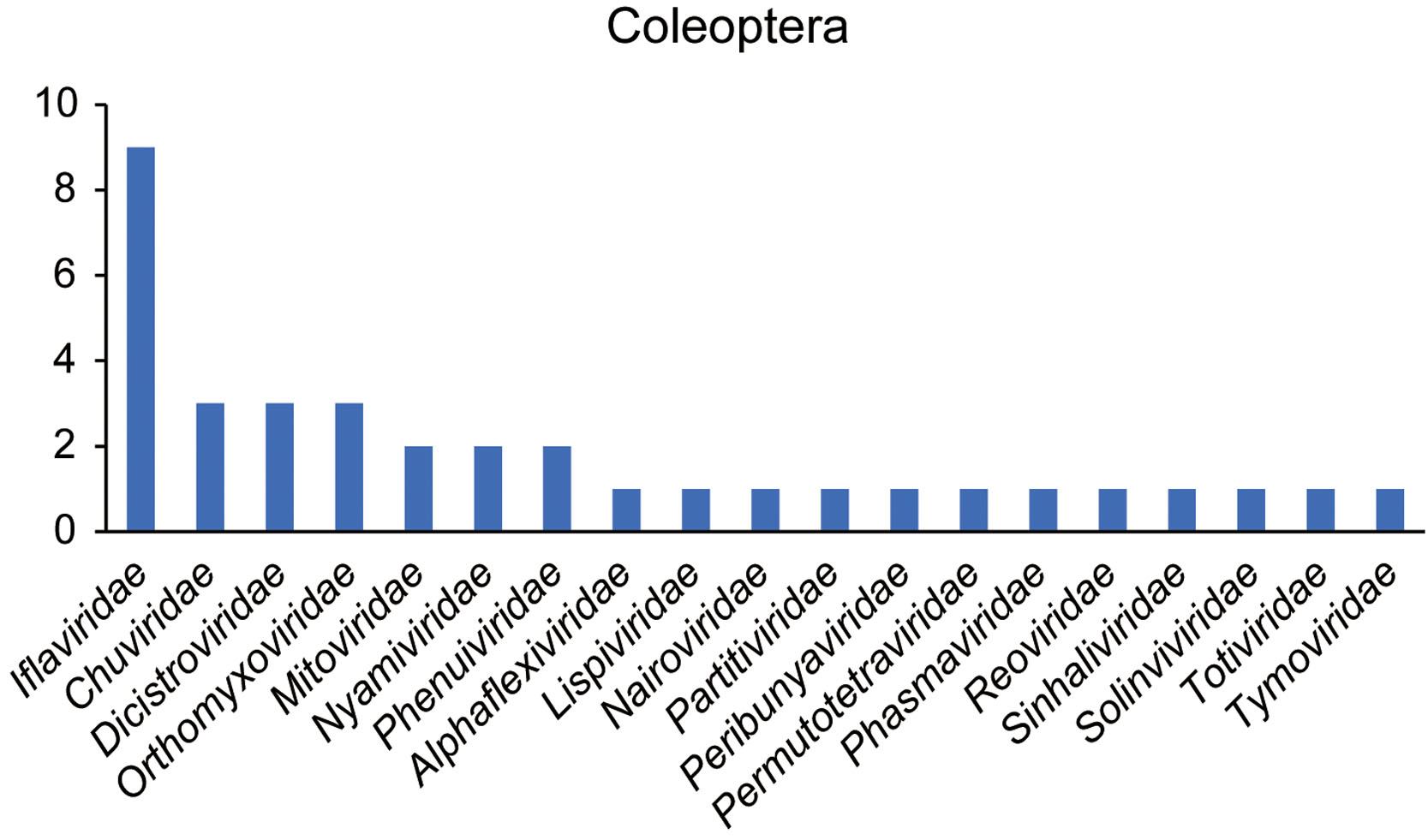

Supplement: Supplementary file 7 — Supplementary Fig. S6. Diversity of RNA viruses in Coleoptera. [file mmc7.jpg]

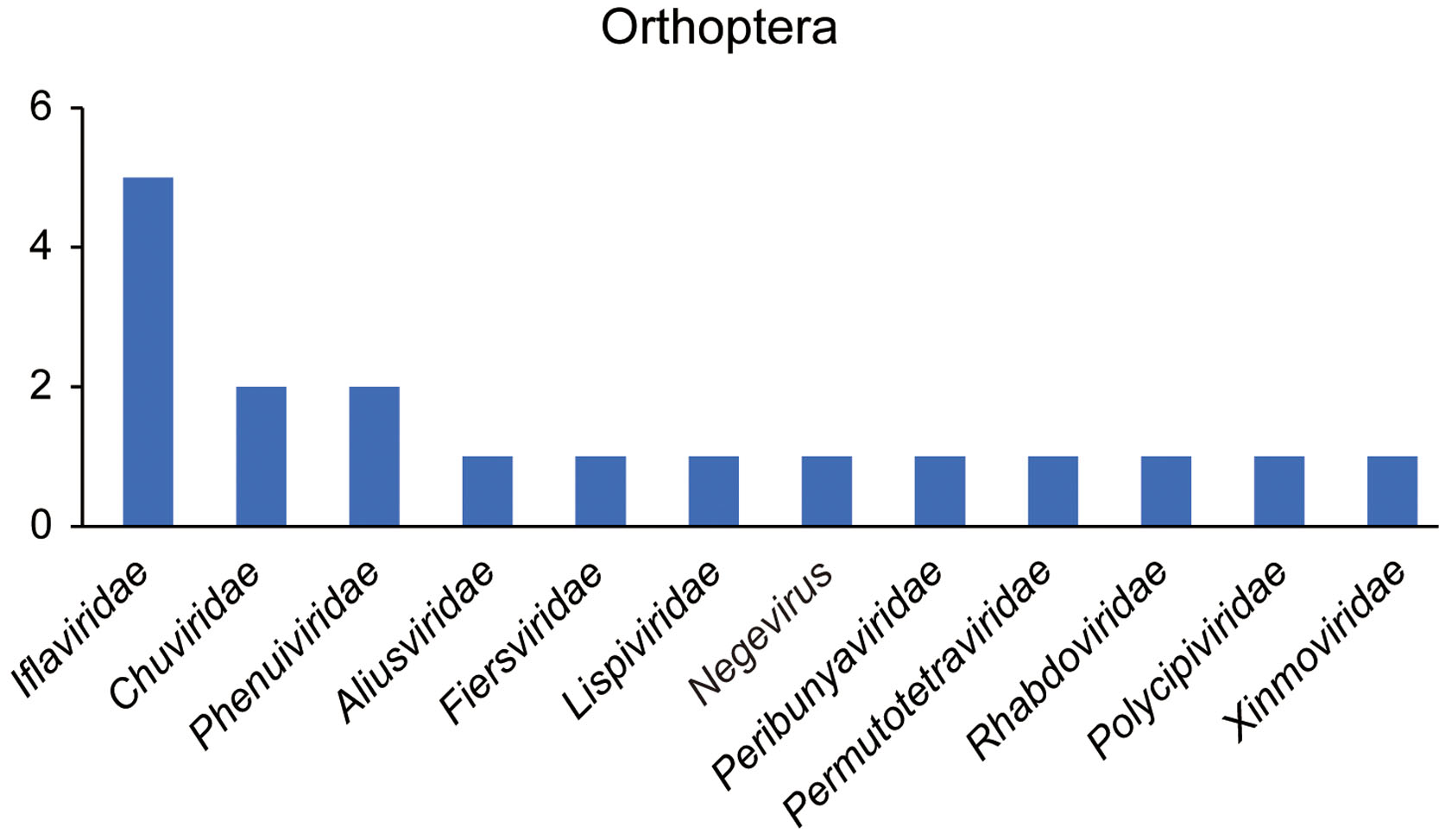

Supplement: Supplementary file 8 — Supplementary Fig. S7. Diversity of RNA viruses in Orthoptera. [file mmc8.jpg]
